# Association between B-type natriuretic peptide and long-term mortality in patients with acute severe hypertension visiting the emergency department

**DOI:** 10.1038/s41598-022-25705-1

**Published:** 2022-12-05

**Authors:** Byung Sik Kim, Yonggu Lee, Young-Hyo Lim, Jinho Shin, Jeong-Hun Shin

**Affiliations:** 1 Division of Cardiology, Department of Internal Medicine, Hanyang University College of Medicine, Hanyang University Guri Hospital, 153 Gyeongchun-ro, Guri, Gyeonggi-do 11923 Republic of Korea; 2 Division of Cardiology, Department of Internal Medicine, Hanyang University College of Medicine, Hanyang University Seoul Hospital, Seoul, Republic of Korea

**Keywords:** Hypertension, Cardiovascular diseases

## Abstract

B-type natriuretic peptide (BNP) is a well-established prognostic factor for cardiovascular disorders. However, the association between BNP levels and mortality in patients with acute severe hypertension remains unclear. This study aimed to investigate the association between BNP levels and long-term mortality in patients with acute severe hypertension visiting the emergency department (ED). This retrospective study included patients aged ≥ 18 years who were admitted to the ED between 2016 and 2019 with acute severe hypertension (systolic blood pressure ≥ 180 mmHg or diastolic blood pressure ≥ 100 mmHg). Patients were categorized into tertiles according to BNP levels upon admission to the ED. Of the 3099 patients with acute severe hypertension, 6.4% in the first (lowest) tertile, 24.8% in the second tertile, and 44.4% in the third (highest) tertile of BNP died within 3-years. After adjusting for clinically relevant variables, patients in the second tertile of BNP (adjusted hazard ratio [HR], 2.64; 95% confidence interval [CI], 1.96–3.55), and patients in the third tertile of BNP (adjusted HR 4.18; 95% CI, 3.09–5.64) had a significantly higher risk of 3-year all-cause mortality than those in the first tertile of BNP. Therefore, BNP may be valuable for the initial assessment to identify high-risk patients among those with acute severe hypertension.

## Introduction

Acute severe hypertension is characterized by an abrupt and severe increase in blood pressure (BP) in patients with or without known hypertension^1^. Acute BP elevation can result in acute progressive injury to the heart, brain, kidney, and the other vasculature^2,3^. These organ injuries are called acute hypertension-mediated organ damage (HMOD), and their presence is a crucial factor for the management and prognosis of acute severe hypertension^4,5^. Although the prognosis of patients with acute severe hypertension has improved in the recent years, their mortality rate remains high^6–8^. Furthermore, despite the high mortality risk, studies on prognostic factors regarding this disease are limited^9–14^.

B-type natriuretic peptide (BNP) is a well-established prognostic marker for various cardiovascular disorders, especially heart failure^15,16^. In addition, BNP levels are associated with an increased risk of mortality in patients with hypertension and in the general population^17,18^. However, there are no data on the association between BNP levels and mortality in patients with acute severe hypertension.

In this study, we assessed the association between BNP levels and long-term mortality in patients with acute severe hypertension visiting the emergency department (ED).

## Methods

### Study participants

**Figure 1:**
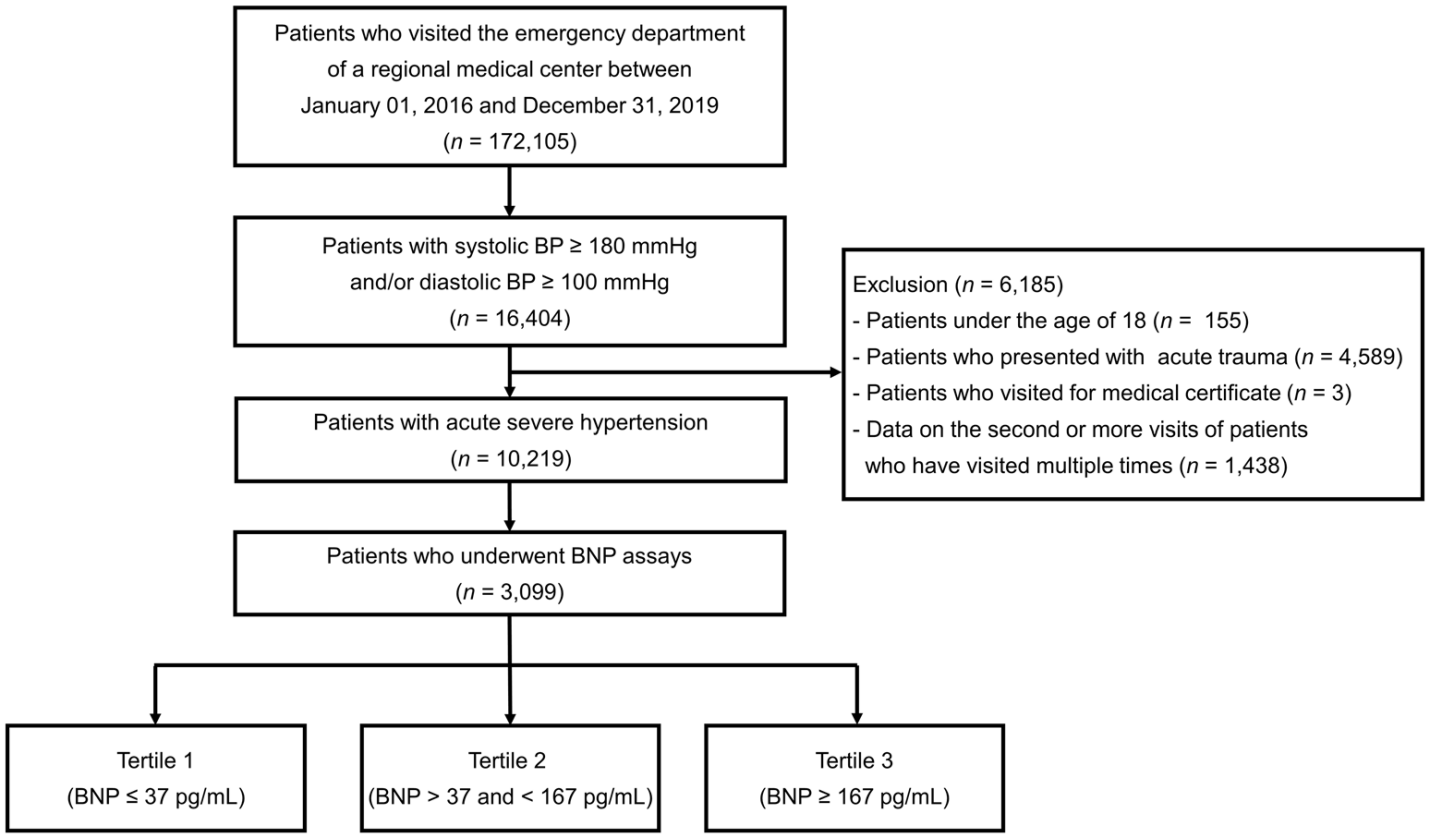
Study flowchart. Among the 10,219 patients with acute severe hypertension, 3099 who underwent a BNP assay were analyzed. The patients were classified into three groups according to the BNP level as follows: tertile 1 (BNP ≤ 37 pg/mL), tertile 2 (37 pg/mL < BNP < 167 pg/mL), and tertile 3 (BNP ≥ 192 pg/mL). BNP, B-type natriuretic peptide.

This retrospective cohort study was conducted in a single regional emergency medical center affiliated with the Academic University Hospital in Guri-si, Gyeonggi-do, Korea. The study design, detailed definitions of comorbidities, and primary results have been published previously^6^. We reviewed the medical records of 172,105 patients who visited the ED between January 2016 and December 2019. In total, 16,404 patients met the blood pressure (BP) criteria for acute severe hypertension, defined as a systolic blood pressure (SBP) ≥ 180 mmHg or diastolic blood pressure (DBP) ≥ 100 mmHg. Patients under 18 years of age, those presenting with acute trauma, or those who visited the certificate were excluded. If the patients visited the ED multiple times, only the first visit was included in this study. Among the 10,219 patients with acute severe hypertension, 3099 who underwent a BNP assay were analyzed (Fig. 1). The study was conducted in accordance with the Declaration of Helsinki and was reviewed and approved by the institutional review board of Hanyang University Guri Hospital. The Institutional Review Board of Hanyang University Guri Hospital waived the requirement for written informed consent.

### B-type natriuretic peptide assay

Plasma BNP levels were measured by immunoassay using a Beckman Coulter DXI 800 instrument (Beckman Coulter Diagnostics, Brea, CA, USA). The patients were classified into three groups according to the BNP level as follows: tertile 1 (BNP ≤ 37 pg/mL), tertile 2 (37 pg/mL < BNP < 167 pg/mL), and tertile 3 (BNP ≥ 167 pg/mL).

### Data collection

Data were collected from the electronic medical records by an experienced data collector under the supervision of the principal investigator. The following demographic and clinical characteristics were extracted: age, sex, initial BP, and traditional cardiovascular risk factors including a history of hypertension, diabetes mellitus, dyslipidemia, chronic kidney disease, end-stage renal disease, cigarette smoking, and alcohol consumption status. History of ischemic stroke, hemorrhagic stroke, heart failure, and coronary artery disease was also recorded. The following laboratory data were obtained: BNP level, estimated glomerular filtration rate (eGFR), troponin-I level, and hemoglobin level. In addition, to evaluate the presence of acute HMOD, diagnostic test findings, such as the urine dipstick test, chest radiography, and electrocardiography (ECG), were obtained.

Additionally, data on events during the index hospitalization and follow-up periods (admission, discharge, ED revisits, readmission, and death) were collected. The incidence and timing of mortality were extracted from the National Health Insurance Service in South Korea, and other event data were extracted from electronic medical records.

### Definition

The presence of acute HMOD was defined as accompaniment of the following conditions: acute heart failure, acute ischemic stroke, acute hypertensive encephalopathy, intracerebral hemorrhage, acute hypertensive retinopathy, acute coronary syndrome, acute renal failure, and acute aortic dissection^6^. Cardiomegaly was diagnosed when the ratio between the maximal horizontal cardiac diameter and the maximal horizontal inner thoracic cage diameter was > 0.5 on chest radiography^19^. Left ventricular hypertrophy (LVH) was diagnosed with ECG findings that satisfied the Sokolow-Lyon criterion (the amplitude of S in V1 plus the amplitude of R in V5 or V6 ≥ 3.5 mV) or the Cornell voltage criterion (the amplitude of R in aVL plus the amplitude of S or QS complex in V3 with a cutoff of > 2.8 mV in men and > 2.0 mV in women)^20^. In the ED, BP was measured in the brachial artery using an automatic BP machine, Spot Vital Signs LXi (Welch Allyn, Skaneateles Falls, NY, USA).

### Statistical analyses

All categorical data were presented as numbers and percentages. Continuous variables were expressed as medians and interquartile ranges because all continuous variables showed a skewed distribution on the Kolmogorov–Smirnov test. Categorical variables were compared using the chi-squared test or Fisher's exact test, whereas continuous variables were compared using the Kruskal–Wallis test followed by Dunn’s multiple comparison test.

Kaplan–Meier survival analyses and log-rank tests were used to compare the cumulative survival probability according to the BNP tertiles. Using the BNP tertile 1 level as a reference, hazard ratios (HRs) and 95% confidence intervals (CIs) for 3-year all-cause mortality according to BNP tertiles were analyzed using multivariate Cox proportional hazards regression analyses, with or without adjustment for other clinically relevant variables. We considered the clinically relevant variables such as, baseline characteristics (age, sex, SBP, DBP, cigarette smoking, and alcohol consumption), comorbidities (hypertension, diabetes mellitus, ischemic stroke, hemorrhagic stroke, coronary artery disease, chronic kidney disease, and end-stage renal disease), and components of HMOD (estimated glomerular filtration rate, proteinuria, cardiomegaly on chest radiography, LVH, and myocardial ischemia on ECG). Variable inflation factors (VIFs) were calculated for these variables and all VIFs were low except for those within the tertiles of the BNP, so multi-collinearity would not be a problem (Supplementary Table S1). Missing rate for candidate variables were reported in Supplementary Table S1. We excluded variables with high missing rates such as, cigarette smoking, alcohol consumption, and proteinuria from the adjusted variables of the Cox proportional hazards regression analyses. We conducted the Cox proportional hazard regression with complete-case analysis, which involves restricting the analysis to individuals with no missing data. The number of subjects included in the fully adjusted Cox proportional hazard regression analyses is 2836. In addition, we performed Cox proportional hazard regression analyses that also included variables with high missing rates after imputing the missing data using multiple imputation. Supplementary Table S2 shows the comparison of variables between the original and imputed data.

Additionally, we performed a subgroup analysis with multivariable Cox proportional hazards regression analysis according to the presence or absence of acute HMOD. We also evaluated the association between BNP levels and 3-year all-cause mortality in patients without LVH on ECG. All tests were two-tailed, and statistical significance was set at p < 0.05. All statistical analyses were conducted using the open-source statistical software R (version 4.1.0, www.R-project.org) and R-studio (version 1.4.1, www.rstudio.com) and statistical packages, including rms, descr, survival, tableone, survminer, car, and amelia.

### Ethical declarations

The study was conducted in accordance with the Declaration of Helsinki and was reviewed and approved by the institutional review board of Hanyang University Guri Hospital (IRB. 2020-01-028). The Institutional Review Board of Hanyang University Guri Hospital waived the requirement for written informed consent.

## Results

### Baseline characteristics

A total of 3099 patients were included, and follow-up data up to 5.2 years were analyzed. The median follow-up period was 2.6 years (interquartile range, 1.4–4.0 years). The baseline characteristics of the patients according to BNP tertile are shown in Table 1. The mean age of the patient was 66.1 years, and less than half (*n* = 1435, 46.3%) were women. Patients with higher tertiles of BNP were older (56 vs. 71 vs. 76, *p* < 0.001) and had a higher proportion of women (34.1% vs. 51.8% vs. 53.0%, *p* < 0.001). Acute HMOD was most frequently observed in patients in the highest tertile of BNP (36.6% vs. 40.6% vs. 66.6%, *p* < 0.001). Patients with higher tertiles of BNP had more cardiovascular risk factors and comorbidities, including hypertension, diabetes mellitus, ischemic and hemorrhagic stroke, coronary artery disease, heart failure, chronic kidney disease, and end-stage renal disease, than patients with the lowest tertile of BNP. Patients with higher tertiles of BNP also had a higher SBP, higher creatinine and troponin-I levels, and lower estimated glomerular filtration rate and hemoglobin levels. In addition, the higher tertiles of the BNP group had more proteinuria, cardiomegaly on chest radiography, and LVH or myocardial ischemia on ECG.

**Table 1:** Baseline characteristics according to tertiles of B-type natriuretic peptide.

	All patients (*n* = 3099)	Tertile 1* (*n* = 1035)	Tertile 2^†^ (*n* = 1031)	Tertile 3^‡^ (*n* = 1033)	*p*-value
Age, median (IQR)	68 (55, 79)	56 (47, 64)^§¶^	71 (59, 80)^↑^	76 (65, 83)	< 0.001
Women, *n* (%)	1435 (46.3)	353 (34.1)	534 (51.8)	548 (53.0)	< 0.001
**Medical history, *n* (%)**					
Hypertension	1827 (60.2)	451 (44.9)	630 (62.5)	746 (72.9)	< 0.001
Diabetes mellitus	913 (30.4)	199 (20.0)	285 (28.7)	429 (42.2)	< 0.001
Dyslipidemia	348 (11.7)	119 (12.0)	130 (13.2)	99 (9.8)	0.061
Ischemic stroke	292 (9.8)	46 (4.6)	116 (11.7)	130 (12.9)	< 0.001
Hemorrhagic stroke	91 (3.1)	17 (1.7)	45 (4.6)	29 (2.9)	0.001
Coronary artery disease	425 (14.2)	85 (8.6)	145 (14.7)	195 (19.3)	< 0.001
Heart failure	222 (7.4)	14 (1.4)	29 (2.9)	179 (17.8)	< 0.001
Chronic kidney disease	321 (10.7)	17 (1.7)	57 (5.8)	247 (24.5)	< 0.001
End-stage renal disease	155 (5.2)	1 (0.1)	11 (1.1)	143 (14.2)	< 0.001
**Social history, *n* (%)**					
Cigarette smoking	595 (25.6)	266 (37.2)	161 (21.1)	168 (19.8)	< 0.001
Alcohol consumption	780 (33.4)	373 (51.8)	219 (28.7)	188 (22.1)	< 0.001
**Triage vitals, median (IQR)**					
SBP, mmHg	185 (166, 201)	180 (160, 194)^§¶^	187 (171, 205)	188 (173, 205)	< 0.001
DBP, mmHg	104 (99, 113)	105 (101, 112)^§¶^	104 (96, 113)	104 (94, 114)	< 0.001
**Laboratory tests, median (IQR)**					
BNP, pg/mL	74 (26, 271)	18 (12, 26)^§¶^	75 (52, 109)^↑^	49 (271, 1171)	< 0.001
Mean serum creatinine, mg/dL	0.87 (0.70, 1.14)	0.80 (0.69, 0.95)^§¶^	0.81 (0.65, 1.06)^↑^	1.10 (0.80, 2.24)	< 0.001
Mean eGFR, mL/min/1.73 m^2^	82 (55, 97)	96 (84, 106)^§¶^	82 (6, 95)^↑^	56 (23, 82)	< 0.001
Troponin-I, ng/mL	0.01 (0.01, 0.04)	0.01 (0.01, 0.01)^§¶^	0.01 (0.01, 0.03)^↑^	0.04 (0.01, 0.08)	< 0.001
Hb, g/dL	13.5 (11.9, 14.9)	14.6 (13.5, 15.6)^§¶^	13.4 (12.1, 14.7)^↑^	12.1 (10.4, 13.7)	< 0.001
**Urinary analysis**					
Proteinuria^a^, *n* (%)	802 (38.3)	99 (17.5)	245 (33.2)	458 (58.2)	< 0.001
Chest radiography					
Cardiomegaly, *n* (%)	459 (15.3)	84 (8.4)	157 (15.7)	218 (21.7)	< 0.001
**Electrocardiography**					
LVH, *n* (%)	336 (11.2)	65 (6.5)	93 (9.3)	178 (17.7)	< 0.001
Myocardial ischemia, *n* (%)	332 (11.0)	82 (8.2)	95 (9.5)	155 (15.4)	< 0.001
Acute HMOD, *n* (%)	1486 (48.0)	379 (36.6)	419 (40.6)	688 (66.6)	< 0.001

Data are presented as *n* (%) or median (IQR), as appropriate. IQR, interquartile range; SBP, systolic blood pressure; DBP, diastolic blood pressure; BNP, B-type natriuretic peptide; eGFR, estimated glomerular filtration rate; Hb, hemoglobin; LVH, left ventricular hypertrophy; HMOD, hypertension-mediated organ damage.

*The range of BNP levels in tertile 1 is ≤ 37 pg/mL.

^†^The range of BNP levels in tertile 2 was between > 37 and < 167 pg/mL.

^‡^The range of BNP levels in tertile 3 is ≥ 167 pg/mL.

^a^Proteinuria was defined as dipstick urinalysis result ≥ 1 +

^§^Post hoc *p*: tertile 1 versus tertile 2: statistically significant, *p* < 0.05.

^¶^Post hoc *p*: tertile 1 versus tertile 3: statistically significant, *p* < 0.05.

^↑^Post hoc *p*: tertile 2 vs. tertile 3: statistically significant *p* < 0.05.

### Outcomes of the index hospitalization and during the follow-up

Of all the patients, 1988 were admitted, and six died in the ED. Patients in the higher tertiles of BNP were more likely to be admitted than those in the lowest tertile of BNP (47.9% vs. 62.5% vs. 82.1%, *p* < 0.001). During the follow-up period, the rates of ED revisit and readmission were not significantly different between the groups, except for the 1-year revisit, which was higher in patients with a higher tertile of BNP. In contrast, the 1-month, 3-month, 1-year, and 3-year mortality rates were higher in patients with higher tertiles of BNP than in those with the lowest tertile of BNP (Table 2).

**Table 2:** Outcomes of the index visit to the emergency department and during the follow-up period according to tertiles of B-type natriuretic peptide.

	All patients (*n* = 3099)	Tertile 1* (*n* = 035)	Tertile 2^†^ (*n* = 1031)	Tertile 3^‡^ (*n* = 1033)	*p*-value
**Outcomes of the index visit to the ED, *n* (%)**					
Admission	1988 (64.1)	496 (47.9)	644 (62.5)	848 (82.1)	< 0.001
Discharge	725 (23.4)	385 (37.2)	255 (24.7)	85 (8.2)	< 0.001
Discharge against medical advice	380 (12.3)	154 (14.9)	132 (12.8)	94 (9.1)	< 0.001
Death in the emergency department	6 (0.2)	0 (0)	0 (0)	6 (0.6)	0.002
**Revisit to ED, *n* (%)**					
1-month revisit	245 (9.52)	84 (9.86)	80 (9.31)	81 (9.40)	0.918
3-months revisit	442 (17.2)	121 (14.2)	154 (17.9)	167 (19.4)	0.014
1-year revisit	785 (30.5)	212 (24.9)	274 (31.9)	299 (34.7)	< 0.001
**Readmission, *n* (%)**					
1-month readmission	167 (6.5)	69 (8.1)	52 (6.0)	46 (5.3)	0.053
3-months readmission	251 (9.7)	84 (9.9)	82 (9.5)	85 (9.8)	0.963
1-year readmission	407 (15.8)	125 (14.7)	147 (17.0)	135 (15.6)	0.397
**Mortality, *n* (%)**					
1-month mortality	192 (6.2)	10 (1.0)	66 (6.4)	116 (11.2)	< 0.001
3-months mortality	302 (9.8)	17 (1.6)	98 (9.5)	187 (18.1)	< 0.001
1-year mortality	531 (17.1)	35 (3.4)	186 (18.0)	310 (30.0)	< 0.001
3-year mortality	781 (25.2)	66 (6.4)	256 (24.8)	459 (44.4)	< 0.001

Data are presented as *n* (%). ED, emergency department.

*The range of BNP levels in tertile 1 is ≤ 37 pg/mL.

^†^The range of BNP levels in tertile 2 was between > 37 and < 167 pg/mL.

^‡^The range of BNP levels in tertile 3 is ≥ 167 pg/mL.

**Figure 2:**
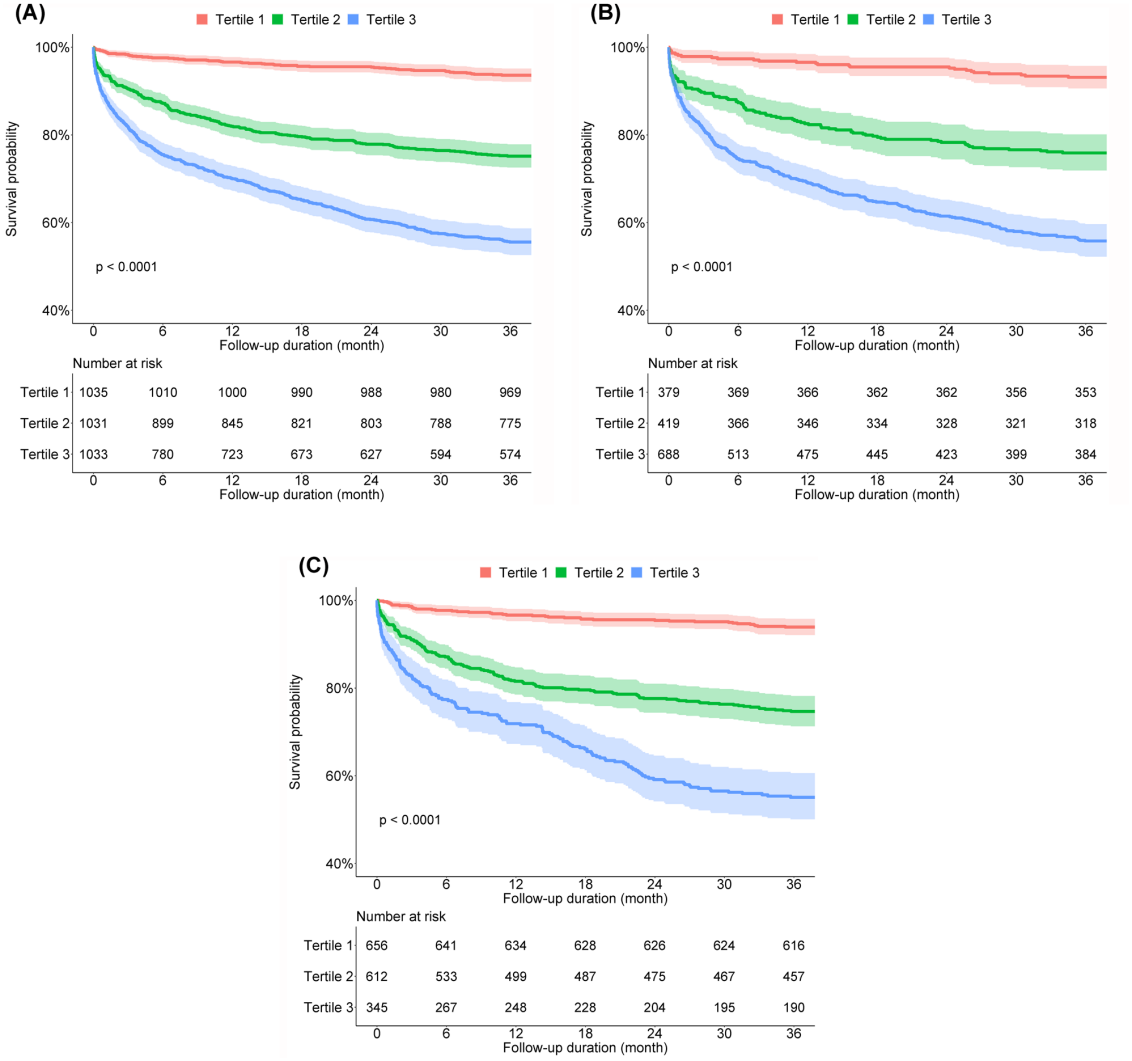
Cumulative survival probability according to tertiles of BNP in (**A**) all patients; (**B**) patients with acute hypertension-mediated organ damage; and (**C**) patients without acute HMOD. The cumulative survival was significantly lower in patients with higher tertiles of BNP. In addition, this trend was consistently observed in the subgroup analysis according to the presence or absence of acute HMOD. BNP, B-type natriuretic peptide; HMOD, hypertension-mediated organ damage.

Based on the BNP levels, the cumulative survival probability was analyzed using the Kaplan–Meier method, and the results are shown in Fig. 2. Among the three groups, cumulative survival was significantly lower in patients with higher tertiles of BNP (Fig. 2A). This trend was consistently observed in the subgroup analysis according to the presence or absence of acute HMOD (Fig. 2B, C). Table 3 shows the independent association between BNP levels and all-cause mortality using Cox proportional hazards regression analyses. Compared with patients in the lowest tertile of BNP, those in higher tertiles of BNP were associated with a higher risk of 3-year all-cause mortality. After adjusting for sex, age, SBP, DBP, cigarette smoking, alcohol consumption, comorbidities, and components of subclinical HMOD, patients in the second tertile of BNP (adjusted HR, 2.64; 95% CI, 1.96–3.55), and patients in the third (highest) tertile of BNP (adjusted HR 4.18; 95% CI, 3.09–5.64) had a significantly higher risk of 3-year all-cause mortality than those in the first tertile of BNP. Among the subgroups with or without acute HMOD, patients in the second and third tertiles of BNP also showed a significantly higher risk of 3-year all-cause mortality than those in the first tertile of BNP. These results were similarly observed when analyzed using imputed data (Supplementary Table S3).

**Table 3:** The hazard ratios for mortality according to tertiles of B-type natriuretic peptide.

	Unadjusted HR (95% CI)	Model 1^a^ (95% CI)	Model 2^b^ (95% CI)	Model 3^c^ (95% CI)	Model 4^d^ (95% CI)
**All patients**					
Tertile 1*	REF	REF	REF	REF	REF
Tertile 2^†^	4.43 (3.38–5.81)	2.62 (1.99–3.47)	2.66 (2.01–3.52)	2.57 (1.93–3.42)	2.64 (1.96–3.55)
Tertile 3^‡^	9.01 (6.96–11.67)	4.60 (3.51–6.04)	4.68 (3.56–6.14)	4.18 (3.15–5.57)	4.18 (3.09–5.64)
**Patients with acute HMOD**					
Tertile 1*	REF	REF	REF	REF	REF
Tertile 2^†^	3.98 (2.59–6.13)	2.28 (1.46–3.54)	2.27 (1.46–3.52)	2.19 (1.40–3.44)	2.16 (1.36–3.42)
Tertile 3^‡^	8.43 (5.64–12.58)	3.79 (2.49–5.77)	3.76 (2.47–5.72)	3.44 (2.22–5.33)	3.24 (2.07–5.10)
**Patients without acute HMOD**					
Tertile 1*	REF	REF	REF	REF	REF
Tertile 2^†^	4.77 (3.37–6.75)	3.02 (2.10–4.35)	3.13 (2.17–4.51)	3.02 (2.07–4.40)	3.24 (2.19–4.81)
Tertile 3^‡^	9.42 (6.65–13.34)	5.44 (3.76–7.86)	5.82 (4.01–8.44)	5.01 (3.38–7.44)	5.24 (3.44–7.99)

HR, hazard ratio; CI, confidence interval; REF, reference.

*The range of BNP levels in tertile 1 is ≤ 37 pg/mL.

^†^The range of BNP levels in tertile 2 was between > 37 and < 167 pg/mL.

^‡^The range of BNP levels in tertile 3 is ≥ 167 pg/mL.

^a^Model 1: Adjusted for age and sex.

^b^Model 2: Adjusted for age, sex, systolic blood pressure, and diastolic blood pressure.

^c^Model 3: Adjusted for age, sex, systolic blood pressure, diastolic blood pressure, and comorbidities (hypertension, diabetes mellitus, ischemic stroke, hemorrhagic stroke, heart failure, coronary artery disease, chronic kidney disease, and end-stage renal disease).

^d^Model 4: Adjusted for age, sex, systolic blood pressure, diastolic blood pressure, comorbidities (hypertension, diabetes mellitus, ischemic stroke, hemorrhagic stroke, heart failure, coronary artery disease, chronic kidney disease, and end-stage renal disease), and components of hypertension-mediated organ damage (estimated glomerular filtration rate, cardiomegaly on chest radiography, left ventricular hypertrophy on electrocardiography, and myocardial ischemia on electrocardiography).

**Figure 3:**
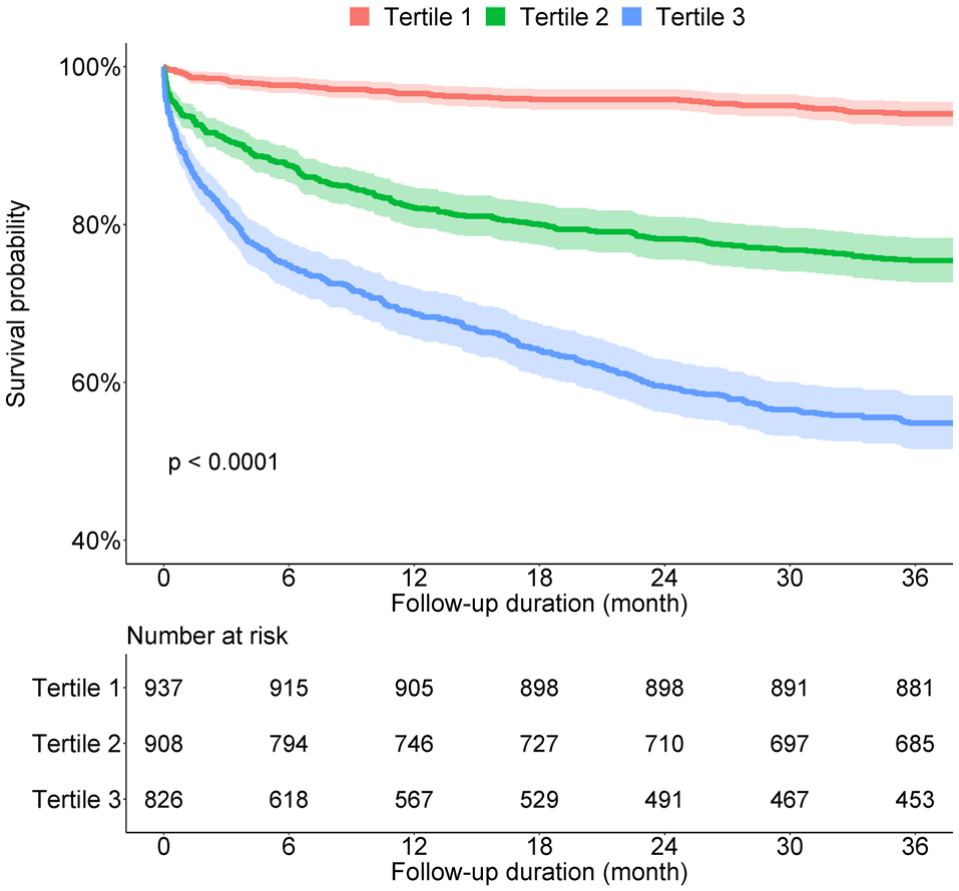
Cumulative survival probability according to tertiles of BNP in patients without LVH on ECG. The cumulative survival was significantly lower in patients with higher tertiles of BNP in patients without LVH on ECG. BNP, B-type natriuretic peptide; LVH, left ventricular hypertrophy; ECG, electrocardiography.

Additionally, we performed a subgroup analysis of patients without LVH on ECG. Figure 3 shows the cumulative survival probability according to the tertiles of BNP in patients without LVH on ECG. Similar to the previous findings, cumulative survival was significantly lower in patients with higher tertiles of BNP. After adjusting for covariates, patients in the second tertile of BNP (adjusted HR 2.86; 95% CI, 2.08–3.93) and patients in the third tertile of BNP (adjusted HR 4.72; 95% CI, 3.42–6.53) had a significantly higher risk of 3-year all-cause mortality than those in the first tertile of BNP (Table 4).

**Table 4:** The hazard ratios for mortality according to tertiles of B-type natriuretic peptide in patients without left ventricular hypertrophy on electrocardiography.

	Unadjusted HR (95% CI)	Model 1^a^ (95% CI)	Model 2^b^ (95% CI)	Model 3^c^ (95% CI)	Model 4^d^ (95% CI)
**Patients without LVH**					
Tertile 1*	REF	REF	REF	REF	REF
Tertile 2^†^	4.66 (3.48–6.25)	2.83 (2.09–3.83)	2.85 (2.13–3.91)	2.80 (2.05–3.83)	2.86 (2.08–3.93)
Tertile 3^‡^	9.86 (7.45–13.07)	5.14 (3.82–6.91)	5.27 (3.91–7.10)	4.74 (3.46–6.48)	4.72 (3.42–6.53)

HR, hazard ratio; CI, confidence interval; LVH, left ventricular hypertrophy; REF, reference.

*The range of BNP levels in tertile 1 is ≤ 37 pg/mL.

^†^The range of BNP levels in tertile 2 was between > 37 and < 167 pg/mL.

^‡^The range of BNP levels in tertile 3 is ≥ 167 pg/mL.

^a^Model 1: Adjusted for age and sex.

^b^Model 2: Adjusted for age, sex, systolic blood pressure, and diastolic blood pressure.

^c^Model 3: Adjusted for age, sex, systolic blood pressure, diastolic blood pressure, and comorbidities (hypertension, diabetes mellitus, ischemic stroke, hemorrhagic stroke, heart failure, coronary artery disease, chronic kidney disease, and end-stage renal disease).

^d^Model 4: Adjusted for age, sex, systolic blood pressure, diastolic blood pressure, comorbidities (hypertension, diabetes mellitus, ischemic stroke, hemorrhagic stroke, heart failure, coronary artery disease, chronic kidney disease, and end-stage renal disease), and components of hypertension-mediated organ damage (estimated glomerular filtration rate, cardiomegaly on chest radiography, and myocardial ischemia on electrocardiography).

## Discussion

The present study showed that BNP levels were an independent risk factor for all-cause mortality in patients with acute severe hypertension. The risk of all-cause mortality tended to increase more in the group with higher BNP levels. In addition, an association between BNP and all-cause mortality was consistently observed in patients with or without acute HMOD as well as in patients without LVH on ECG.

In response to volume or pressure overload, the cardiac tissue releases a natriuretic peptide (NP); as such, increased levels of NP reflect the wall stress of the heart^21^. In this regard, NP has a high diagnostic accuracy in discriminating heart failure from other conditions in patients presenting with acute dyspnea^22^. Therefore, clinical practice guidelines recommended that NP should be measured in all patients presenting with symptoms suggestive of heart failure^23,24^. In addition to its diagnostic value in heart failure, NP can also aid in the risk stratification of patients with cardiovascular disorders such as myocardial infarction, valvular heart disease, and pulmonary embolism^15,16^. The prognostic value of NP has also been reported in the general population. Wang et al.^18^ reported that BNP levels above 20.0 pg/mL in men and 23.3 pg/mL in women were associated with a 1.62-fold increased risk of mortality in the general population. In a previous study of patients with hypertension, patients with N-terminal pro-brain natriuretic peptide ≥ 50.8 pg/mL had a 1.99-fold increased risk of mortality, and those with N-terminal pro-brain natriuretic peptide ≥ 133 pg/mL had a 3.3-fold increased risk of mortality^17^. Similar to previous studies regarding other diseases, our study also showed a 2.64-fold increased risk of mortality in patients in the second tertile of BNP whose BNP levels were only above 37.0 pg/mL.

Several potential mechanisms could explain these findings. First, patients with higher tertiles of BNP have more traditional cardiovascular risk factors and comorbidities, such as old age, hypertension, diabetes mellitus, chronic kidney disease, stroke, coronary artery disease, heart failure, and a higher frequency of proteinuria, cardiomegaly, and abnormal ECG findings than those with the lowest tertile of BNP. However, the association between BNP level and the risk of mortality was consistently observed even after adjusting for confounding factors. Second, given the mechanism of BNP secretion, a higher BNP level may reflect a higher cardiac load. A previous study reported that increased BNP levels were associated with left ventricular diastolic dysfunction and hypertrophy in patients with hypertension^25^. Our study also showed a higher frequency of cardiomegaly on chest radiography and LVH on ECG in patients with higher tertiles of BNP. Third, a previous study suggested that BNP level reflects silent heart disease, such as LVH, systolic and diastolic dysfunction, left atrial enlargement, and myocardial ischemia in asymptomatic patients^26^. Therefore, patients with higher BNP levels are more likely to have undiagnosed heart disease or structural changes. Future studies are needed to clarify the underlying mechanism of the association between BNP levels and the risk of mortality in these populations.

Increased BNP levels are associated with established heart failure, LVH, and subclinical cardiac damage. Phelan et al.^27^ reported that increased BNP levels can reflect subclinical cardiac remodeling and inflammation in asymptomatic patients with hypertension. In our study, a significant association between BNP levels and the risk of mortality persisted in the subgroup of patients without acute HMOD. Moreover, this association was consistently observed in patients without LVH on the ECG. These results suggest that BNP is an associated factor for adverse outcomes, even in patients who do not develop hypertensive cardiac remodeling. Therefore, increased BNP level may be a potential indicator of subclinical HMOD in patients with acute severe hypertension. In this respect, more detailed assessment with the BPN assay can be recommended to check the presence of cardiovascular disease or asymptomatic organ damage that may affect the treatment of hypertension and to predict high-risk groups that require intensive care and appropriate follow-up strategies.

This study had several limitations. First, it was a retrospective study; therefore, data on the baseline characteristics from the electronic medical records were insufficient compared with the data from prospective studies. For example, the missing rates for some variables were relatively high. Second, because this was a single-center study, our results may not be applicable to other clinical settings. Third, given the observational nature of this study, there are limitations in interpreting causal relationships. Fourth, we only used baseline laboratory results, including BNP levels, and did not consider the follow-up results. Fifth, diagnostic tests, including the BNP levels were not measured in all patients, and it is likely that the BNP test was performed in relatively high-risk patients, so the possibility of selection bias could not be excluded (Supplementary Table S4). Finally, as the National Health Insurance Service did not provide the cause of mortality, we could not identify cardiovascular mortality or adverse cardiovascular events such as myocardial infarction and stroke. However, the data on the incidence of all-cause mortality and date of mortality were accurate because data from the National Health Insurance Service encompasses the entire Korean population.

## Conclusions

The present study demonstrated that BNP levels are an independent risk factor for long-term mortality in patients with acute severe hypertension. Moreover, an association between BNP and all-cause mortality was consistently observed, even in patients without LVH on ECG. BNP may be valuable for the initial assessment to identify a high risk of mortality among patients with acute severe hypertension. Further studies are required to determine the optimal screening and treatment strategies for cardiovascular events or deaths in patients with acute severe hypertension, with respect to the BNP levels.

## Supplementary Information

Supplementary Tables.

## Data Availability

The datasets generated the current study are available from the corresponding author on reasonable request.
