# Supplementary material for: Association between B-type natriuretic peptide and long-term mortality in patients with acute severe hypertension visiting the emergency department
Source: Sci Rep. 2022 Dec 5;12:21001. doi: 10.1038/s41598-022-25705-1 (PMC9722913; doi:10.1038/s41598-022-25705-1)
Supplement: Supplementary file 1 — Supplementary Tables. [file 41598_2022_25705_MOESM1_ESM.docx]

| **Supplementary Table S1.** Missing rate and variance inflation factors for potential covariates of Cox proportional hazards regression analyses | | |  |
| --- | --- | --- | --- |
| Potential covariates for Cox proportional hazards regression analyses | Missing value, n (%) | Variance inflation factors | |
| BNP | 0 | Tertile 2: 4.38  Tertile 3: 4.78 | |
| Age | 0 | 1.56 | |
| Sex | 0 | 1.33 | |
| SBP | 0 | 1.08 | |
| DBP | 0 | 1.12 | |
| Cigarette smoking | 773 (24.9) | 1.40 | |
| Alcohol consumption | 767 (24.7) | 1.39 | |
| Hypertension | 63 (2.0) | 1.15 | |
| Diabetes mellitus | 93 (3.0) | 1.25 | |
| Ischemic stroke | 108 (3.5) | 1.08 | |
| Hemorrhagic stroke | 114 (3.7) | 1.03 | |
| Heart failure | 113 (3.6) | 1.20 | |
| Coronary artery disease | 105 (3.4) | 1.05 | |
| Chronic kidney disease | 111 (3.6) | 1.70 | |
| End-stage renal disease | 114 (3.7) | 1.28 | |
| eGFR | 20 (0.6) | 2.00 | |
| Proteinuria | 1,007 (32.5) | 1.25 | |
| Cardiomegaly on chest radiography | 96 (3.1) | 1.05 | |
| Left ventricular hypertrophy on ECG | 92 (3.0) | 1.09 | |
| Myocardial ischemia on ECG | 92 (3.0) | 1.06 | |

BNP, B-type natriuretic peptide; SBP, systolic blood pressure; DBP, diastolic blood pressure; eGFR, estimated glomerular filtration rate; LVH, left ventricular hypertrophy; ECG, electrocardiography.

| **Supplementary Table S2.** Comparison between the original data and imputed data | | | | |  |
| --- | --- | --- | --- | --- | --- |
| Potential covariates for Cox proportional hazards regression analyses | Original data  (n = 3,099) | Imputed data  (n = 3,099) | *p*-value^*^ | SMD | |
| Age, median (IQR) | 68 (55, 79) | 68 (55, 79) | 1 | <0.001 | |
| Women, *n* (%) | 1,435 (46.3) | 1,435 (46.3) | 1 | <0.001 | |
| SBP, mmHg, median (IQR) | 185 (166, 201) | 185 (166, 201) | 1 | <0.001 | |
| DBP, mmHg, median (IQR) | 104 (99, 113) | 104 (99, 113) | 1 | <0.001 | |
| Cigarette smoking, *n* (%) | 595 (25.6) | 764 (24.7) | 0.565 | 0.029 | |
| Alcohol consumption, *n* (%) | 780 (33.4) | 1003 (32.4) | 0.417 | 0.023 | |
| Hypertension, *n* (%) | 1,827 (60.2) | 1,860 (60.0) | 0.92 | 0.003 | |
| Diabetes mellitus, *n* (%) | 913 (30.4) | 937 (30.2) | 0.929 | 0.003 | |
| Ischemic stroke, *n* (%) | 292 (9.8) | 293 (9.5) | 0.716 | 0.01 | |
| Hemorrhagic stroke, *n* (%) | 91 (3.1) | 93 (3.0) | 0.973 | 0.003 | |
| Heart failure, *n* (%) | 222 (7.4) | 224 (7.2) | 0.795 | 0.008 | |
| Coronary artery disease, *n* (%) | 425 (14.2) | 429 (13.8) | 0.72 | 0.01 | |
| Chronic kidney disease, *n* (%) | 321 (10.7) | 327 (10.6) | 0.841 | 0.006 | |
| End-stage renal disease, *n* (%) | 155 (5.2) | 158 (5.1) | 0.914 | 0.004 | |
| eGFR, mL/min/1.73 m^2^, median (IQR) | 82 (55, 97) | 82 (56, 97) | 0.994 | <0.001 | |
| Proteinuria, *n* (%) | 802 (38.3) | 1,124 (36.3) | 0.138 | 0.043 | |
| Cardiomegaly on chest radiography, *n* (%) | 459 (15.3) | 469 (15.1) | 0.898 | 0.004 | |
| LVH on ECG, *n* (%) | 336 (11.2) | 345 (11.1) | 0.992 | 0.001 | |
| Myocardial ischemia on ECG, *n* (%) | 332 (11.0) | 336 (10.8) | 0.835 | 0.006 | |

SMD, standardized mean differences; IQR, interquartile range; SBP, systolic blood pressure; DBP, diastolic blood pressure; eGFR, estimated glomerular filtration rate; LVH, left ventricular hypertrophy; ECG, electrocardiography.

^*^Categorical variables were compared using the chi-squared test or Fisher's exact test, whereas continuous variables were compared using the Mann–Whitney *U* test.

| **Supplementary Table S3**. The hazard ratios for mortality according to tertiles of B-type natriuretic peptide using imputed data   \|  \| Unadjusted HR  (95% CI) \| Model 1^a^  (95% CI) \| Model 2^b^  (95% CI) \| Model 3^c^  (95% CI) \| Model 4^d^  (95% CI) \| \| --- \| --- \| --- \| --- \| --- \| --- \| \| All patients \|  \|  \|  \|  \|  \| \| Tertile 1^*^ \| REF \| REF \| REF \| REF \| REF \| \| Tertile 2^†^ \| 4.43 (3.38-5.81) \| 2.62 (1.99-3.47) \| 2.60 (1.96-3.44) \| 2.55 (1.92-3.37) \| 2.41 (1.81-3.19) \| \| Tertile 3^‡^ \| 9.01 (6.96-11.67) \| 4.60 (3.51-6.04) \| 4.60 (3.50-6.04) \| 4.25 (3.22-5.62) \| 3.71 (2.78-4.95) \| \| Patients with acute HMOD \|  \|  \|  \|  \|  \| \| Tertile 1^*^ \| REF \| REF \| REF \| REF \| REF \| \| Tertile 2^†^ \| 3.98 (2.59-6.13) \| 2.28 (1.46-3.54) \| 2.31 (1.48-3.59) \| 2.24 (1.44-3.49) \| 2.10 (1.34-3.27) \| \| Tertile 3^‡^ \| 8.43 (5.64-12.58) \| 3.79 (2.49-5.77) \| 3.77 (2.48-5.75) \| 3.48 (2.27-5.35) \| 2.91 (1.88-4.51) \| \| Patients without acute HMOD \|  \|  \|  \|  \|  \| \| Tertile 1^*^ \| REF \| REF \| REF \| REF \| REF \| \| Tertile 2^†^ \| 4.77 (3.37-6.75) \| 3.02 (2.10-4.35) \| 3.00 (2.08-4.33) \| 2.93 (2.03-4.24) \| 2.78 (1.92-4.02) \| \| Tertile 3^‡^ \| 9.42 (6.65-13.34) \| 5.44 (3.76-7.86) \| 5.86 (4.04-8.50) \| 5.30 (3.61-7.80) \| 4.92 (3.30-7.34) \| |
| --- | --- | --- | --- | --- | --- | --- | --- | --- | --- | --- | --- | --- | --- | --- | --- | --- | --- | --- | --- | --- | --- | --- | --- | --- | --- | --- | --- | --- | --- | --- | --- | --- | --- | --- | --- | --- | --- | --- | --- | --- | --- | --- | --- | --- | --- | --- | --- | --- | --- | --- | --- | --- | --- | --- | --- | --- | --- | --- | --- | --- | --- | --- | --- | --- | --- | --- | --- | --- | --- | --- | --- | --- | --- | --- | --- | --- | --- | --- |

HR, hazard ratio; CI, confidence interval; REF, reference.

^a^Model 1: Adjusted for age and sex.

^b^Model 2: Adjusted for age, sex, systolic blood pressure, diastolic blood pressure, cigarette smoking, and alcohol consumption.

^c^Model 3: Adjusted for age, sex, systolic blood pressure, diastolic blood pressure, cigarette smoking, alcohol consumption, and comorbidities (hypertension, diabetes mellitus, ischemic stroke, hemorrhagic stroke, heart failure, coronary artery disease, chronic kidney disease, and end-stage renal disease).

^d^Model 4: Adjusted for age, sex, systolic blood pressure, diastolic blood pressure, cigarette smoking, alcohol consumption, comorbidities (hypertension, diabetes mellitus, ischemic stroke, hemorrhagic stroke, heart failure, coronary artery disease, chronic kidney disease, and end-stage renal disease), and components of hypertension-mediated organ damage (estimated glomerular filtration rate, proteinuria, cardiomegaly on chest radiography, left ventricular hypertrophy on electrocardiography, and myocardial ischemia on electrocardiography).

^*^The range of BNP levels in tertile 1 is ≤ 37 pg/mL.

^†^The range of BNP levels in tertile 2 was between > 37 and < 167 pg/mL.

^‡^The range of BNP levels in tertile 3 is ≥ 167 pg/mL.

| **Supplementary Table S4.** Comparison of included and excluded participants | | | |  |
| --- | --- | --- | --- | --- |
|  | Included  (n = 3,099) | Excluded  (n = 7,120) | *p*-value | |
| Age, median (IQR) | 68 (55, 79) | 54 (43, 66) | <0.001 | |
| Women, *n* (%) | 1,435 (46.3) | 3,394 (47.7) | 0.212 | |
| Medical history, *n* (%) |  |  |  | |
| Hypertension | 1,827 (60.2) | 2,679 (39.5) | <0.001 | |
| Diabetes mellitus | 913 (30.4) | 1,215 (18.1) | <0.001 | |
| Dyslipidemia | 348 (11.7) | 572 (8.6) | <0.001 | |
| Ischemic stroke | 292 (9.8) | 345 (5.2) | <0.001 | |
| Hemorrhagic stroke | 91 (3.0) | 154 (2.3) | 0.047 | |
| Coronary artery disease | 425 (14.2) | 306 (4.6) | <0.001 | |
| Heart failure | 222 (7.4) | 93 (1.4) | <0.001 | |
| Chronic kidney disease | 321 (10.7) | 243 (3.7) | <0.001 | |
| End-stage renal disease | 155 (5.2) | 120 (1.8) | <0.001 | |
| Social history, *n* (%) |  |  |  | |
| Cigarette smoking | 595 (25.6) | 1001 (26.4) | <0.001 | |
| Alcohol consumption | 780 (33.4) | 1561 (39.7) | <0.001 | |
| Triage vitals, median (IQR) |  |  |  | |
| SBP, mmHg | 185 (166, 201) | 174 (159, 188) | <0.001 | |
| DBP, mmHg | 104 (99, 113) | 104 (100, 112) | 0.026 | |
| Laboratory tests done, *n* (%) | 3,094 (99.8) | 5,293 (74.3) | <0.001 | |
| Serum creatinine, mg/dL, median (IQR) | 0.87 (0.70, 1.14) | 0.79 (0.64, 0.96) | <0.001 | |
| eGFR, mL/min/1.73 m^2^, median (IQR) | 82.0 (55.3, 97.0) | 96.00 (79.0, 108.0) | <0.001 | |
| Troponin-I, ng/mL, median (IQR) | 0.01 (0.01, 0.04) | 0.01 (0.01, 0.01) | <0.001 | |
| Hb, g/dL, median (IQR) | 13.5 (11.9, 14.9) | 14.1 (12.9, 15.3) | <0.001 | |
| Urinary analysis done, *n* (%) | 2,091 (67.5) | 3,386 (47.6) | <0.001 | |
| Proteinuria^a^, *n* (%) | 802 (38.3) | 820 (24.2) | <0.001 | |
| Chest radiography done, *n* (%) | 3,011 (97.2) | 5,017 (70.5) | <0.001 | |
| Cardiomegaly, *n* (%) | 459 (15.3) | 528 (10.0) | <0.001 | |
| Electrocardiography done, *n* (%) | 3,014 (97.3) | 4,251 (59.7) | <0.001 | |
| LVH, *n* (%) | 336 (11.2) | 413 (9.7) | 0.054 | |
| Myocardial ischemia, *n* (%) | 332 (11.0) | 150 (3.5) | <0.001 | |
| Acute HMOD, *n* (%) | 1,486 (48.0) | 1,020 (14.3) | <0.001 | |
| Outcomes of the index ED visit, *n* (%) |  |  |  | |
| Admission | 1,988 (64.1) | 2,149 (30.2) | <0.001 | |
| Discharge | 725 (23.4) | 4,389 (61.6) | <0.001 | |
| Discharge against medical advice | 380 (12.3) | 579 (8.1) | <0.001 | |
| Death in the emergency department | 6 (0.2) | 3 (0.0) | 0.044 | |
| Revisit to ED, *n* (%) |  |  |  | |
| 1-month revisits | 245 (9.5) | 480 (9.5) | 0.955 | |
| 3-month revisit | 442 (17.2) | 796 (15.7) | 0.098 | |
| 1-year revisit | 785 (30.5) | 1384 (27.2) | 0.003 | |
| Readmission, *n* (%) |  |  |  | |
| 1-month readmission | 167 (6.5) | 289 (5.7) | 0.184 | |
| 3-month readmission | 251 (9.7) | 422 (8.3) | 0.041 | |
| 1-year readmission | 407 (15.8) | 612 (12.0) | <0.001 | |
| Mortality, *n* (%) |  |  |  | |
| 1-month mortality | 192 (6.2) | 116 (1.6) | <0.001 | |
| 3-month mortality | 302 (9.8) | 187 (2.6) | <0.001 | |
| 1-year mortality | 531 (17.1) | 370 (5.2) | <0.001 | |
| 3-year mortality | 781 (25.2) | 637 (8.9) | <0.001 | |

Data are presented as n (%) or median (IQR), as appropriate. IQR, interquartile range; SBP, systolic blood pressure; DBP, diastolic blood pressure; BNP, B-type natriuretic peptide; eGFR, estimated glomerular filtration rate; Hb, hemoglobin; LVH, left ventricular hypertrophy; HMOD, hypertension-mediated organ damage.

^a^Proteinuria was defined as dipstick urinalysis result ≥ 1+.
